# Evaluating Osteogenic Potential of Ligamentum Flavum Cells Cultivated in Photoresponsive Hydrogel that Incorporates Bone Morphogenetic Protein-2 for Spinal Fusion

**DOI:** 10.3390/ijms161023318

**Published:** 2015-09-28

**Authors:** Chih-Wei Chiang, Wei-Chuan Chen, Hsia-Wei Liu, I-Chun Wang, Chih-Hwa Chen

**Affiliations:** 1 Bone and Joint Research Center, Department of Orthopedics and Traumatology, Taipei Medical University Hospital, School of Medicine, College of Medicine, Taipei Medical University, Taipei 110, Taiwan; E-Mails: kiwi8502017@gmail.com (C.-W.C.); itispay@gmail.com (W.-C.C.); 2 Graduate Institute of Biomedical Electronics and Bioinformatics, National Taiwan University, Taipei 106, Taiwan; 3 Department of Life Science, College of Science and Engineering, Fu Jen Catholic University, New Taipei City 242, Taiwan; E-Mail: 079336@mail.fju.edu.tw; 4 Department of Orthopedic Surgery, Chang Gung Memorial Hospital, Keelung 204, Taiwan; 5 College of Medicine, Chang Gung University, Taoyuan 333, Taiwan; 6 Graduate Institute of Biomedical Materials and Tissue Engineering, College of Biomedical Engineering, Taipei Medical University, Taipei 110, Taiwan

**Keywords:** Ligamentum flavum, tissue engineering, spinal fusion, PEGDA hydrogels, BMP-2

## Abstract

Regenerative medicine is increasingly important in clinical practice. Ligamentum flava (LF) are typically removed during spine-related surgeries. LF may be a source of cells for spinal fusion that is conducted using tissue engineering techniques. In this investigation, LF cells of rabbits were isolated and then characterized by flow cytometry, morphological observation, and immunofluorescence staining. The LF cells were also cultivated in polyethylene (glycol) diacrylate (PEGDA) hydrogels that incorporated bone morphogenetic protein-2 (BMP-2) growth factor, to evaluate their proliferation and secretion of ECM and differentiation *in vitro*. The experimental results thus obtained that the proliferation, ECM secretion, and differentiation of the PEGDA-BMP-2 group exceeded those of the PEGDA group during the period of cultivation. The mineralization and histological staining results differed similarly. A nude mice model was utilized to prove that LF cells on hydrogels could undergo osteogenic differentiation *in vivo*. These experimental results also revealed that the PEGDA-BMP-2 group had better osteogenic effects than the PEGDA group following a 12 weeks after transplantation. According to all of these experimental results, LF cells are a source of cells for spinal fusion and PEGDA-BMP-2 hydrogel is a candidate biomaterial for spinal fusion by tissue engineering.

## 1. Introduction

Regenerative medicine is being increasingly used in clinical practice. Functional tissues have recently been obtained *in vitro* and regenerated *in vivo*. Bone tissue engineering is an emerging field in orthopedic research and clinical practice. Ligamentum flava (LF) easily degenerate and cause iatrogenic injury, causing back pain and nerve dysfunction. This process is regarded as pathophysiological mechanisms in spine-related syndromes. LF are removed in most spine-related surgeries. Many reports have claimed that LF can ossify under certain conditions, such as treatment by parathyroid hormone [1]. Moreover, spinal fusion is considered to be an important means of treating various spinal disorders. Osteoinductive and/or osteoconductive agents are extensively utilized to induce solid spinal fusion. However, LF has never been seriously used to be a cell source for spinal fusion. Therefore, we believe that the combination of LF cells and tissue engineering can have a role in spinal fusion.

Bone morphogenetic proteins (BMPs) are pleiotropic, osteoinductive proteins of the transforming growth factor-beta (TGF-β) family. BMP-induced signal transduction is an important positive regulator of osteoblastic growth and differentiation [2,3]. BMPs act locally and must, therefore, be delivered directly to the site of regeneration using a carrier matrix [4,5,6]. They can potentially repair local skeletal defects with the formation of new bone from undifferentiated stem cells in association with an osteoconductive carrier. Numerous studies have provided evidence that LF is affected by BMPs. Hayasi *et al.* suggested that BMPs may promote endochondral ossification of LF at ectopic ossification sites [7]. Kim *et al.* developed a treatment that combined BMP-2 and Runx2 to provide osteoblastic differentiation than treatment with either component alone [8]. Yang *et al.* proved that human LF cells that are transduced with Ad/BMP-2 express the osteogenic phenotype and bone nodule formation [9]. Moon *et al.* showed that in human LF cells, BMP-2 considerably up-regulates the expression of osteogenic phenotypes and induces the formation of bone nodules [2].

Photo-responsive hydrogels are investigated for several tissue engineering applications, including the prevention of thrombosis, postoperative adhesion, drug delivery, and cell transplantation, because they can be formed *in situ* in a minimally invasive manner. Photo-polymerized hydrogels, which form three-dimensional networks with high permeability and good biocompatibility, are attractive for use in cell encapsulation and tissue engineering applications [10]. Poly(ethylene glycol) diacrylate (PEGDA)-based polymers that act as scaffolds for cell transplantation and tissue growth have recently become a realistic objective. Ingavle *et al.* showed that PEGDA copolymerized agarose by physical entrapment within agarose and methacrylated CS, improving cellular performance in cartilage tissue engineering [11]. Lu *et al.* transplanted oligodendrocyte progenitor cells with PEGD-based polymers *in vitro*, improving survival of the cells and oligodendrogenic differentiation [12].

Based on the above, in this work BMP-2 is incorporated into a PEGDA network (PEGDA-BMP-2 hydrogel) to improve the proliferation, ECM secretion and differentiation of LF cells *in vitro* to provide an alternative means of spinal fusion in spine tissue engineering. An *in vivo* study is also conducted to evaluate the mineralization potential of PEGDA based hydrogel.

## 2. Results

### 2.1. The in Vitro Study of Ligamentum Flavum Cells

**Figure 1:**
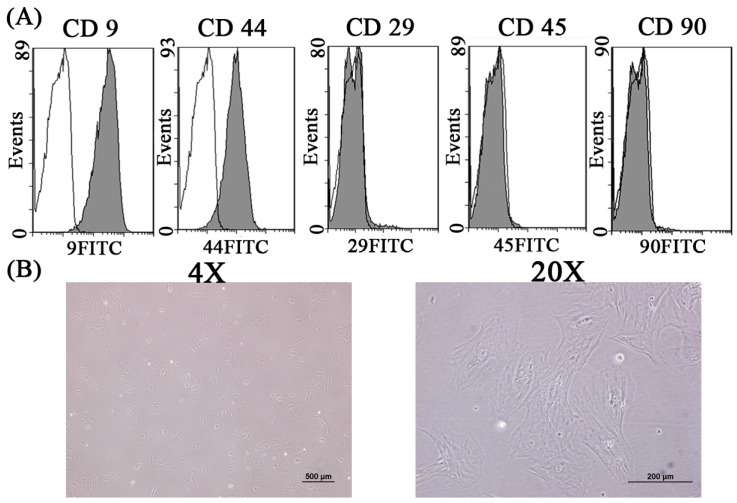
The identification of Ligamentum flavum cells by flow cytometery (**A**) and the morphology of LF cells cultivated on culture dish (**B**).

**Figure 2:**
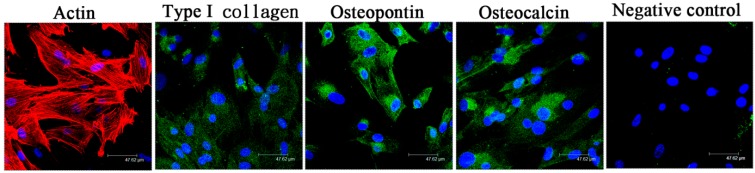
Immunofluorescence staining of Actin, type I collagen, osteopontin, osteocalcin, and negative control for LF cells cultivated on culture dish.

In this study, rabbit LF cells were identified by flow cytometry and by morphological observation. The experimental results herein reveal that around 90% LF cells were positive for CD9 or CD44, whereas fewer than 5% were positive for CD29, CD45, and CD90 (Figure 1A). Analytical results revealed that LF expressed CD9 and CD44, but not CD29, CD45 or CD90. Rabbit LF cells were spindle-shaped (Figure 1B), and maintained this shape for four passages. The phenotype of these LF cells was similar to that of LF cells in humans. Immunofluorescence staining provided evidence of isolated LF cells with osteogenetic properties. As revealed by immunofluorescence staining, the major type of collagen for osteogenesis, type I collagen, was substantially expressed following (cultivation of LF osteocalcin and osteopontin (specific markers of osteogenic-related protein) were similarly expressed after cultivation) (Figure 2).

**Figure 3:**
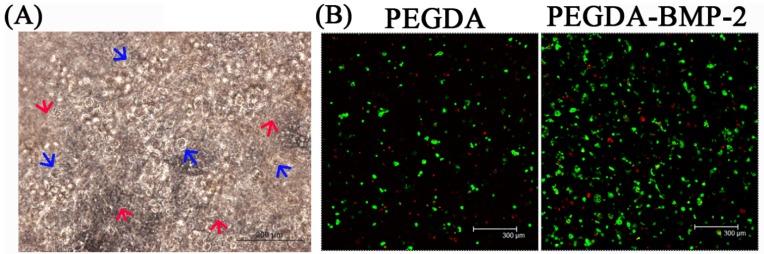
(**A**) Inverted light microscopy image (original magnification, 100×) of 0-day construct immediately after photoencapsulation of LF cells. Blue arrows: cells; red arrows: hydrogel; and (**B**) The LIVE/DEAD staining of LF cells cultivated into PEGDA or PEGDA-BMP-2 hydrogels for fourteen days by bioreactor culture after UV exposure.

The LF cells herein were further cultivated with PEGDA and PEGDA-BMP-2 hydrogels in a rotating wall vessel bioreactor to stimulate osteogenic differentiation, to evaluate cell viability, histology, Alkaline phosphatase (ALP) activity, and cell proliferation, and to study the osteogenic effect of BMP-2 growth factor. Observations of the PEGDA-BMP-2 hydrogel cultivated with LF cells by inverted microscopy immediately after photoencapsulation indicated round cells evenly dispersed throughout the constructs (Figure 3A). After photo-encapsulation, and following 14 days of cultivation in a bioreactor, cell viability was observed by LIVE/DEAD staining. The cell population in the PEGDA-BMP-2 group (around 509 ± 32 of live cells and 115 ± 12 of dead cells, around 81% viability) exceeded that in the PEGDA group (around 171 ± 11 of live cells and 127 ± 16 of dead cells, around 57% viability) (Figure 3B), and more cells were alive (green) than were dead (red).

**Figure 4:**
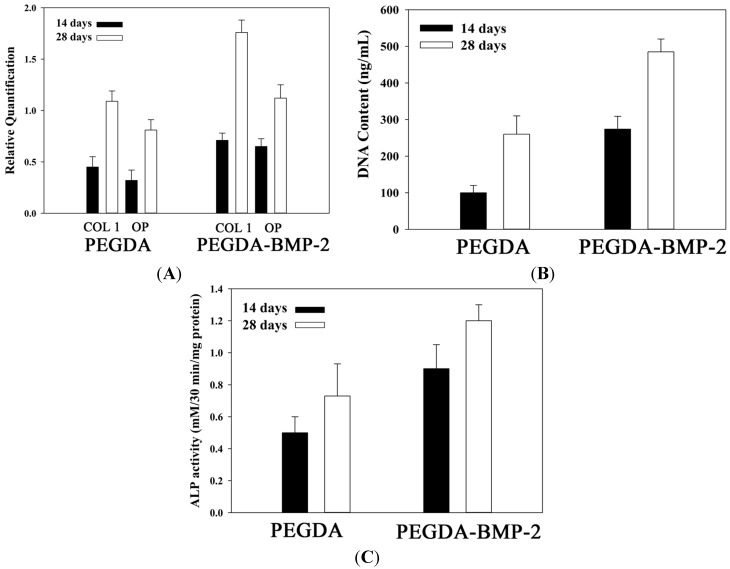
Quantitative analyses of osteogenic gene expressions (**A**); cell proliferation (**B**), and alkaline phosphates secretion (**C**) of LFs cultivated in PEGDA and PEGDA-BMP-2 photo-responsive hydrogels. Triplicates were used for each experiment. For gene expressions of COL I and OP after 28 days cultivation, PEGDA-BMP-2 group showed higher than PEGDA group (*p* < 0.05). For cell proliferation after 28 days cultivation, PEGDA-BMP-2 group showed higher than PEGDA group (*p* < 0.05). For ALP secretion, PEGDA-BMP-2 group showed higher than PEGDA group (*p* < 0.05).

To evaluate the osteogenic potential of LF cells that are cultivated in either PEGDA or PEGDA-BMP-2 hydrogel, osteogenic-related gene expressions, cell proliferation, ALP secretion rate, and histology were analyzed. Type I collagen (COL I) is the major extracellular matrix in bone tissue. Osteopontin (OPN) is the specific marker that regulates normal mineralization within the extracellular matrices of bones and up-regulates at sites of pathologic and ectopic calcification. Accordingly, the osteogenic potential of LF cells was evaluated using two genes. For the analysis of osteogenic-related gene expressions, the PEGDA-BMP-2 group exhibited better osteogenic expressions than did the PEGDA group in the cultivation period. As shown in Figure 4, the osteogenic-related gene expressions of type I collagen (COL I) and osteopontin (OP) were up-regulated above those of the PEGDA group (*p* < 0.05). According to the experimental results, BMP-2 growth factor had an osteogenic effect on LF cells. As well as affecting gene expressions, BMP-2 growth factor affected the proliferation (Figure 4B) and the ALP secretion rate (Figure 4C) of LF cells. The DNA content, an indicator of LF cell proliferation, revealed better cell proliferation in the PEGDA-BMP-2 group than in the PEGDA group during the cultivation period (*p* < 0.05). The activity of ALP, a marker protein in bone, was higher in the PEGDA-BMP-2 group than in the PEGDA group during the cultivation period (*p* < 0.05).

**Figure 5:**
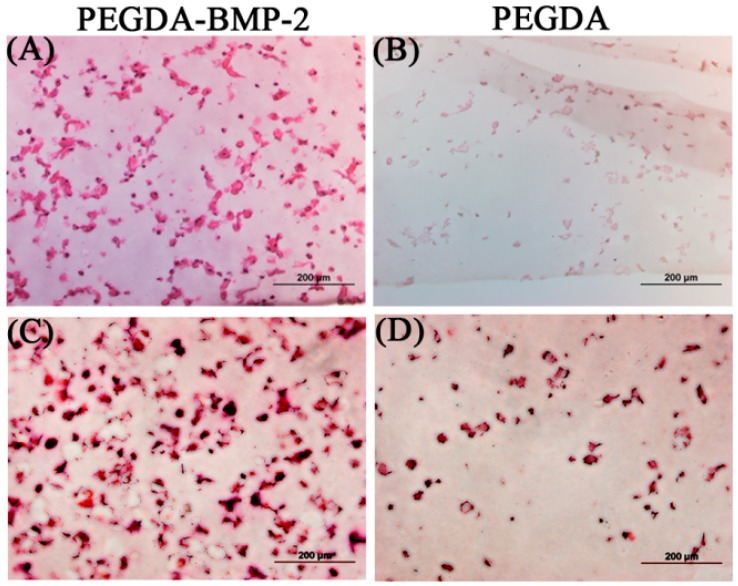
Representative H&E stain (**A**,**B**) and Alizarin Red S stain (**C**,**D**) of PEGDA-BMP-2 (**A**,**C**) and PEGDA (**B**,**D**) photo-responsive hydrogels containing LFs after 28 days of culture. Scale bar = 200 μm.

The cells and hydrogels in this study were also examined by histological analysis (Figure 5). Alizarin Red S staining has been used for decades to evaluate calcium-rich deposits by cells in culture. Based on the histological results in Figure 5, H&E and Alizarin Red S staining were consistent with the results concerning DNA content and ALP activity during the cultivation period. The staining revealed that LF cells cultivated that were in the PEGDA-BMP-2 group exhibited higher proliferation and secreted more ALP than those in the PEGDA group.

**Figure 6:**
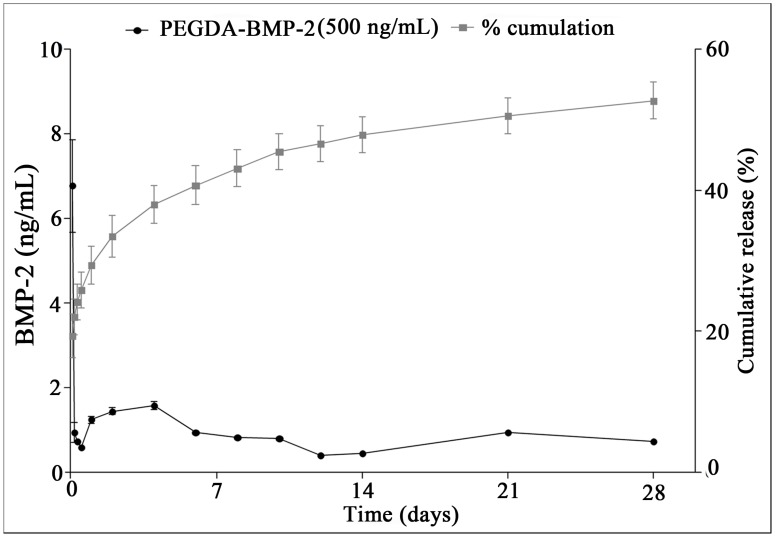
Release profile of bone morphogenetic protein-2 (BMP-2) from 500-ng/mL PEGDA-BMP-2 scaffold on enzyme-linked immunosorbent assay. Black circle graphs showed noncumulative release after each time point. Gray rectangle graphs show the percentage cumulative release. Triplicates were used for each experiment.

PEGDA-BMP-2 scaffold initially loaded with 500 ng/mL of the BMP-2. On ELISA, PEGDA-BMP-2 scaffolds released small amounts of BMP-2 every day for up to 28 days. After 2 h from the release, cumulative release of the 500 ng/mL PEGDA-BMP-2 scaffold was 19% of the original amount, increasing to 54% after 28 days (shown in Figure 6).

### 2.2. The in Vivo Study of LF Cells on Hydrogels

**Figure 7:**
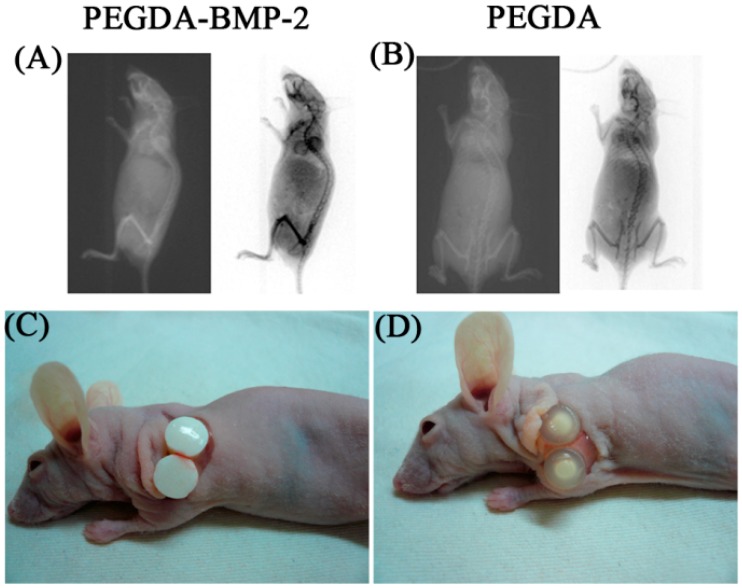
Radiographic assessment of nude mice model and gross morphology of LFs cultivated in PEGDA-BMP-2 (**A**,**C**) and PEGDA (**B**,**D**) photo-responsive hydrogels after 12-week transplantation.

The experimental results concerning *in vitro* evaluation revealed that LF cells that were cultivated in PEGDA or PEGDA-BMP-2 hydrogels underwent osteogenic differentiation. Accordingly, LF cells were shown to undergo osteogenic differentiation both on hydrogels and in the nude mice model *in vivo*. The X-ray absorptiometry are used primarily to evaluate bone mineral density in terms of the status of osteogenesis. X-ray absorptiometry results showed that osteogenesis in the PEGDA-BMP-2 group exceeded that in the PEGDA group (Figure 7A). Twelve weeks after transplantation, the nude mice were sacrificed and the implants were harvested for further evaluation. The experimental results associated with the gross morphology, including elasticity, transparency, and (white) color of the two groups, revealed that the PEGDA-BMP-2 group secreted more proteins than the PEGDA group (Figure 7B).

**Figure 8:**
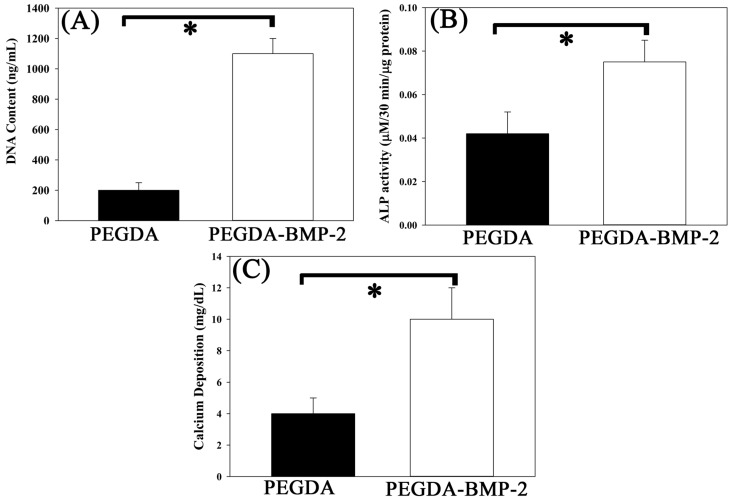
Quantitative analyses of cell proliferation (**A**); alkaline phosphates secretion (**B**) and calcium deposition (**C**) of LFs cultivated in PEGDA and PEGDA-BMP-2 photo-responsive hydrogels after 12-week transplantation. Triplicates were used for each experiment. * *p* < 0.05.

Apart from the gross morphology, cell proliferation, ALP activity, calcium content, and histology were also evaluated (Figure 8). The experimental results concerning cell proliferation *in vivo* were consistent with those *in vitro* (Figure 8A). The *in vivo* and *in vitro* experimental results concerning ALP activity and calcium content (*p* < 0.05) (Figure 8B,C) also showed a similar trend. These experimental results indicated again that the PEGDA-BMP-2 group exhibited better osteogenic effects than the PEGDA group 12 weeks after transplantation (*p* < 0.05). Accordingly, BMP-2 had a critical role in causing LF cells to becoming osteoblast or osteoblast-like cells.

**Figure 9:**
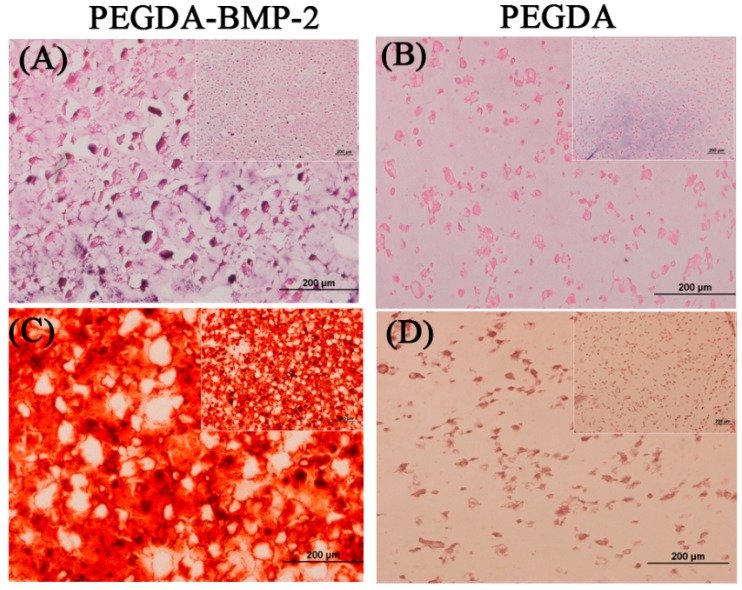
Representative H&E stain (**A**,**B**), Alizarin red stain (**C**,**D**) of PEGDA-BMP-2 (**A**,**C**), and PEGDA (**B**,**D**) photo-responsive hydrogels containing LFs after 12-week transplantation. Scale bar = 200 μm.

The experimental results in Figure 9 include the histological results, including those of H&E staining and Alizarin Red S staining. The lower magnification images were put on the upper right area (Figure 9). According these staining results, significant differences on cell proliferation and calcium deposition were shown either in PEGDA or PEGDA-BMP-2 hydrogel. In addition, the H&E staining results of higher magnification images revealed that LF cells in PEGDA-BMP-2 hydrogel exhibited greater proliferation than those in PEGDA hydrogel 12 weeks after transplantation (Figure 9A). Furthermore, more calcium was deposited by Alizarin Red S stain was deposited in the PEGDA-BMP-2 group than in the PEGDA group in that period (Figure 9B). The experimental results concerning Alizarin Red S staining were consistent with the quantification of calcium content measurement (Figure 8).

## 3. Discussion

Clinically, lumbar spinal stenosis results from hypertrophy of the facet joints and ligamentum flavum [13,14]. Bone morphogenetic protein-2 and transformer growth factor-β1 (TGF-β1) are crucial in the process of ossification of the LF. Progenitor cells in the ligament will then undergo the proliferation, cartilage production, and ossification under the influence of BMP-2 and TGF-β1 [15,16].

The combination of BMP-2 and a biomaterial to achieve a drug delivering behavior needed to control the 3D configuration of BMP-2 for the increase of bone mineral density. BMP-2 could be incorporated onto the surface of the PEGDA hydrogel and retained its activity after covalent conjugation. PEGDA cross-linked BMP-2 could last a sustaining signal better than did soluble BMP-2, and it sustained excellent osteogenic expression *ex vivo*. Based on the osteogenic property, this study tried to develop a 3D system for LF cultivation in tissue engineering.

The identification of LF cells by flow cytometry is seldom discussed. Most relevant studies identify LF cells from their morphology and osteogenic proteins and osteogenic expressions. Zhong *et al.* reported that the morphology of LF cells varied widely, ranging from thin, spindle-shaped cells to polygonal cells or oval-shaped cells at confluence they also observed the expression of type I collagen in LF cells [17]. Specchia *et al.* found that LF cells expressed high levels of ALP and generated a matrix that was rich in type I and III collagen, fibronectin, and osteonectin [1]. The experimental results herein concerning the spindle-shaped morphology of LF cells, and proteins of type I collagen, osteocalcin, and osteopontin support these claims. Therefore, the LF cells that were isolated in this study were consistent with those described in the literature. The analyses of cluster of differentiation (CD) markers in this study may also provide a reference for future research into LF cells.

An appropriate microenvironment will facilitate cell growth and ECM secretion. Cell differentiation varies with the cultivation microenvironment both *in vitro* and *in vivo* [18]. However, the combination of LF cells and scaffolds has never been reported upon in relation to ligament tissue engineering. Many studies have claimed that PEGDA-based hydrogels can provide spaces for cell proliferation [19,20]. Hassan *et al.* showed that PEGDA-based hydrogel for cultivating hADSCs secretes pro-angiogenic growth factors with low cytotoxicity [19]. Sharma *et al.* found that the deposition of cartilage-specific ECM in PEGDA hydrogel and the stimulation of adjacent cartilage tissue were promoted by mesenchymal stem cells *in vitro* [20]. The experimental results herein also reveal that LF cells can proliferate in PEGDA-based microenvironments. Moreover, various studies have shown that BMP-2 growth factor profoundly affects the behaviors of mesenchymal stem cells and osteoblasts, including cell proliferation and differentiation [21,22]. Our previous study demonstrated that BMP-2-hydrogel that was cultivated with periosteum progenitor cells markedly accelerated tendon-bone healing by the neo-formation of fibrocartilage [21]. Olabisi *et al.* suggested that microencapsulation (the incorporation of PEGDA hydrogels) protects cells and prolongs and spatially distributes transgene expression; this process, therefore, can substantially improve gene therapeutic approaches to bone repair [22].

This study reveals that LF cells exhibit osteogenic activity *in vivo*. The abnormal ossification of LF cells in humans is an important cause of thoracic myelopathy. The pathogenesis of spontaneous ossification of LF *in vivo* and the differentiation of normal and abnormal LF are still undergoing experimental research being experimentally examined. Hou *et al.* ossified rat LF cells by embedding recombinant BMP-2 in their epidural space [23]. They found that pathological changes in ossification involved ligament degeneration, cartilage formation and, finally, bone formation. Spinal cord compression reduced the amount of white matter and neurons nine weeks after operation below those in a sham surgery group. Yang *et al.* utilized human LF cells that were transfected by an adenovirus with BMP-2 and implanted into rat abdomens [9]. These LF cells strongly expressed BMP-2, osteocalcin mRNA, and osteocalcin protein. The LF tissue exhibited osteogenesis, but the control group exhibited no bone formation. In earlier studies, LF cells exhibited osteogenic potential and osteogenesis under BMP-2 stimulation. The same result was obtained herein.

BMPs are a family of the TGF-β superfamily that plays an important role in the development of mammalian skeletons [24,25]. BMPs regulate the anabolic and catabolic cycles of bone by affecting osteoblasts, chondrocytes and osteoclasts [9,26]. Of all BMPs, BMP-2 is thought to have a strong anabolic effect on bone formation *in vivo* [27,28]. Chen *et al.* proved that the injection of BMP-2 subcutaneously into the calvaria of fetal rat induced periosteal bone formation without a prior cartilage phase [29]. Their experimental results showed increased expression of bone cell differentiation marker genes, ALP, type I collagen, osteocalcin, osteopontin, and bone sialoprotein. Luppen *et al.* showed BMP-2 restored mineralization by glucocorticoid-inhibited MC3T3-E1 osteoblast cultures [30] and may be functionally related to the accompanying rescue of the differentiation-related cell cycle [31]. Similarly, based on our experimental results, PEGDA-BMP-2 group stimulated DNA content of LF and increased ALP activity and calcium deposition in nude mice model. This would suggested a role for BMP-2 in the promotion and maintenance on bone formation. To our knowledge, soluble BMP-2 might be diffused into the medium less to affect the cell expressions, whereas PEGDA cross-linked BMP-2 in the hydrogel showed the direct effect to influence them. The PEGDA cross-linked BMP-2 with a porous 3D structure to express the slow release property demonstrated that this photocross-linked hydrogel present a non-diffusion-based release style. Chemical cross-linking of BMP-2 expresses the potential for prolonging BMP-2 effect on cell differentiation to further enhance osteoblast differentiation, functionality, and mineralization. BMP-2 affected the osteogenic markers such as ALP and calcium deposition and the gene expressions of OP and COL I in *in vitro* and *ex vivo* study. These results indicated that the LF cells affected by BMP-2 retention in the hydrogel displayed in a functional status in the process of osteogenetic differentiation. An important issue in the 3D microenvironment is the cellular affinity between a biomaterial and cell attachment. In addition, the distribution of cells is crucial for cell growth and differentiation. Consistent stimulation by prolonged BMP-2 retention enhanced the migration and proliferation of LF cells. Cellular proliferation on PEGDA-BMP-2 hydrogel was significantly better than PEGDA hydrogel in the end of the cultivation in *in vitro* study. Furthermore, histological staining, ALP activity, and calcium deposition were higher in PEGDA-BMP-2 hydrogel than in PEGDA hydrogel in *ex vivo* study.

However, BMP-2 has had limited clinical application because it degrades rapidly *in vitro* to less than its working concentration in the microenvironment of the bone system [32]. Therefore, several works have focused on maintaining a low release rate of bioactive BMP-2 over a desired period. Numerous biomaterials are utilized to deliver BMP-2, including hydroxyapatite composite, β-tricalcium phosphate, and hyaluronic acid [33,34]. No research is being conducted to compare the various biomaterials that are utilized with BMP-2 to evaluate their osteogenetic functions *in vivo*. The results herein revealed that PEGDA gel not only preserved BMP-2 function in the microenvironment but also was not toxic to LF cells. Therefore, we suggest that further research into the osteogenetic potential of LF cells should be conducted based on our study model.

Currently, only one recombinant BMP-2 is available for clinical use: rhBMP-2 (Infuse; Medtronic Sofamor Danek, Memphis, TN, USA). Numerous clinical reports have noted that rhBMP-2 promotes bone healing in spine and long bone surgery [35,36]. However, the clinical safety of rhBMP-2 remains a concern. Chan *et al.* found that the use of rhBMP-2 in both acute traumatic or and post-traumatic reconstructive surgery may increase post-operative serous wound discharge [35]. The bone union rate was high in the rhBMP-2 group. The infection and re-operation rates did not significantly differ. Lykissas *et al.* the use of rhBMP-2 to autograft or allograft in lateral lumbar interbody fusion in a retrospective cohort-controlled study, the result of which provided evidence that the rate of post-operative neurologic deficit and the anterior thigh pain in the rhBMP-2 group exceeded those in the allograft or autograft group [37]. Although the surgical approach to lateral lumbar interbody fusion may cause lumbosacral plexus injury, the higher rate of immediate post-operative and persistent neurologic deficit in the rhBMP-2 group revealed that rhBMP-2 may directly injure neurons. All of the complications of rhBMP-2 are associated with BMP-induced immune modulated inflammatory response. Meyer *et al.* verified the safety of using rhBMP-2 in a dog dural membrane [38]. However, the concentration of in their experiments that involved dogs was significantly less than that used clinically. We believe that a higher concentration of rhBMP-2 would cause more complications. The combination of PEDGA and rhBMP-2 could decrease local rhBMP-2 concentration and decrease neurologic sequel. Our experiment also proved that PEDGA and rhBMP-2 could make rhBMP-2 efficient in the bone healing.

## 4. Experimental Section

### 4.1. Isolation of Ligamentum Flavum Cells

All animal procedures were conducted according to the Guide for the Care and Use of Laboratory Animals and were approved by the Committee of Experimental Animal Sciences of Chang Gung Memorial Hospital (identification code: 2010090801, 9 September 2010). LF tissue was obtained from male New Zealand white rabbits (weight, 3.0 kg). The rabbits were anesthetized by intermuscular injection of ketamine (Sigma, St. Louis, MO, USA) and xylazine (Bayer, Kyonggi-do, Korea). Then the LF tissue specimens were harvested and washed with phosphate buffer solution (PBS, Sigma). The dissected specimens were minced into small pieces and then digested in type I collagenase (0.1 mg/mL, Sigma) at 37 °C in 5% CO_2_. After a 3-h digestion, the cells were harvested and cultivated in a 75T flask with Dulbecco’s Modified Eagle Medium (DMEM) containing fetal bovine serum (10% *v*/*v*, FBS, Gibco, Carlsbad, CA, USA) and 1% antibiotics at 5% CO_2_ and 37 °C in a humidified atmosphere.

### 4.2. Identification of Ligamentum Flavum Cells

Identification of LF cells was determined by flow cytometry. The LF cells were harvested after confluence. The LF cells were collected and incubated for 30 min with fluorescein isothiocyanate (FITC)-conjugated antibodies CD 9, CD29, CD44, CD45, and CD90 (Sigma). Then, the LF cells were collected again and analyzed by flow cytometry (Epics XL-MCL; Beckman Coulter, Miami, FL, USA).

### 4.3. Conjugation of Polyethylene Glycol Tethered BMP-2

Lyophilized BMP-2 purchased from R&D Systems (R&D Systems, Abingdon, UK) was reconstituted with sterile 4-mM HCl containing 0.1% bovine serum albumin. Polyethylene glycol (PEG) was reacted with an equimolar amount of acrylate-PEG-*N*-hydroxysuccinimide and BMP-2 (500 ng/mL, Sigma) in NaHCO_3_ buffer (Sigma) to harvest ACRL-PEG-BMP-2 conjugates. The conjugates were further purified by gel filtration with a Sephadex G-25 (J.T. Baker, Phillipsburg, NJ, USA) desalting column, then lyophilized and stored at −70 °C. The PEG-BMP-2 conjugate retained 90% of the BMP-2 for reaction efficacy of binding BMP-2 to PEG.

### 4.4. Gel Fabrication and LF Cells Photo-Encapsulation

**Figure 10:**
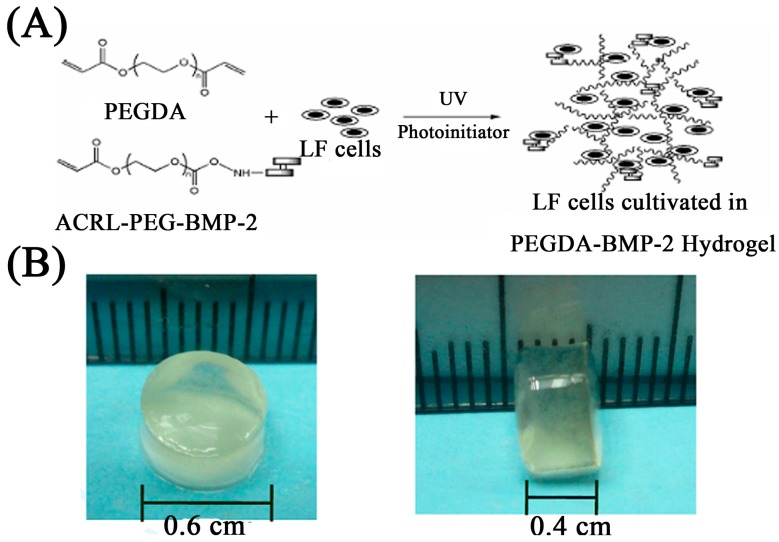
The scheme of LF cells cultivated in photo-responsive hydrogel (**A**) and size (**B**), of photo-responsive hydrogel using in this study.

Hydrogel solution was prepared by mixing 20% (*w*/*v*) PEGDA (PEGDA, *M*_W_ 3400 Da) in sterile PBS with 1% antibiotics (Sigma). The photo-initiator, Irgacure 2959 (Sigma), was added to the PEGDA solution and mixed thoroughly to make a final concentration of 0.15% (*w*/*v*). The ACRL-PEG-BMP-2 conjugates (500 ng/mL) were mediated to the PEGDA solutions of the experimental groups to be 20% (*w*/*v*) mixtures (PEDGA-BMP-2 group). The PEGDA-BMP-2 released small amounts of BMP-2 every day for up to 28 days. The LF cells were re-suspended in the polymer solution to make a concentration of 5 × 10^5^ cells per hydrogel. A 70-μL aliquot of cell-polymer-photo-initiator suspension was loaded into disk-shaped molds with a 6-mm diameter (height = 4 mm), followed by 365 nm UV light (intensity of 10 mW/cm^2^) to gelate the cell polymer constructs for 100 s. The hydrogel constructs were removed from the molds, washed with sterile PBS containing 1% penicillin-streptomycin (Sigma), and incubated in 24-well plates (Figure 10).

### 4.5. In Vitro Release Kinetics of BMP-2

*In vitro* release of BMP-2 from PEGDA-BMP-2 scaffolds was measured with an enzyme-linked immunosorbent assay (ELISA) kit (R&D Systems). The scaffolds were placed in a 24-well plate with 1 mL of DMEM containing 10% FBS and incubated at 37 °C in 5% CO_2_ at 95% humidity. The supernatant was removed and replaced with fresh DMEM solution every two days for up to 28 days. Supernatant samples were kept at −208 °C until they were used for BMP-2 determination. ELISA was performed according to the manufacturer’s protocol. Light absorbance was read with a microreader (MRX II; Dynex Technologies, Chantilly, VA, USA) at a wavelength of 450 nm.

### 4.6. Bioreactor Cultivation in Vitro

All cell-hydrogel constructs were incubated at 37 °C in a rotating bioreactor culture system (Synthecon, Houston, TX, USA) filled with osteogenic medium (including 10 mM β-glycerophosphate (Sigma), 10^−8^ M dexamethasone (Sigma) and 50 μg/mL ascorbic acid (Sigma)) in 5% CO_2_ for up to one and two weeks. The vessel turned at 20 rpm to ensure constant tumbling of the constructs. Half of the medium was replaced every three days. The hydrogel constructs without culture were the contrast. Cell viability, mineralization, collagen content, alkaline phosphatase activity (ALP activity, Sigma), and DNA content (Sigma) were estimated for osteogenesis assay.

### 4.7. In Vitro Release Profile of BMP-2

*In vitro* release of BMP-2 from PEGDA-BMP-2 scaffolds was measured with an enzyme-linked immunosorbent assay (ELISA) kit (R&D Systems). The scaffolds were placed in a 24-well plate with 1 mL of DMEM containing 10% FBS and incubated at 378 °C in 5% CO_2_ at 95% humidity. The supernatant was removed and replaced with fresh DMEM solution every two days for up to 28 days. Supernatant samples were kept at 208 °C until they were used for BMP-2 determination. ELISA was performed according to the manufacturer’s protocol. Light absorbance was read with a microreader (MRX II; Dynex Technologies, Chantilly, VA, USA) at a wavelength of 450 nm.

### 4.8. In Vivo Implantation: Nude Mice Model

Eighteen male nude mice (four weeks old) were purchased from BioLASCO Taiwan Co., Ltd. (Taipei, Taiwan). All experimental protocols were approved by the Animal Care and Experiment Committee of Chang Gung Memorial Hospital at Keelung. Anesthesia was achieved with intramuscular injection of Zoletil 50 (20 mg/kg, Vibrac, Carros, France) and xylazine hydrochloride 0.3 mL/kg (Bayer Korea, Gyeonggi-do, Korea). Under aseptic conditions, separated subcutaneous pockets were created through horizontal incisions and blunt dissections. Cell-hydrogel constructs were then implanted subcutaneously in the dorsa of the nude mice for *in vivo* study. Nude mice were sacrificed at 12 weeks and samples of cell-hydrogel constructs were harvested.

### 4.9. Radiographic Assessment

After euthanization at postoperative 12 weeks, the cell-hydrogel constructs embedded in the backs of the nude mice were examined with X-ray absorptiometry to evaluate the change in osteogenesis.

### 4.10. Cell Viability

Cell viability after photo-encapsulation was observed with a LIVE/DEAD viability/cytotoxicity kit (Molecular Probes, Eugene, OR, USA), following the standard protocols. The constructs were followed biopsy under a confocal microscope (Leica TCS SP5, Leica Microsystems Heidelberg GmbH, Mannheim, Germany).

### 4.11. Alkaline Phosphatase Activity and Cell Proliferation

The ALP activity of cell-hydrogel constructs in the rotating culture system was evaluated at the 14-day and 28-day cultivation. Before measuring ALP activity, the cell-hydrogel constructs were washed with PBS to prevent the serum effect. After the prescribed interval, the cell-hydrogel constructs were lysed and analyzed for ALP activity and total protein, as described previously. Lyophilized scaffolds were digested in 1 mL of a papainase solution (125 mg/mL papain, 100 mM PBS, 10 mM cysteine, and 10 mM ethylenediaminetetraacetic acid, pH 6.3; Worthington Biomedical, Lakewood, NJ, USA) at 60 °C for 18 h. The measurement of ALP activity followed the standard protocols. The total protein content of each lysate was measured using a commercial assay kit (Pierce Biotechnology, Rockford, IL, USA), according to the manufacturer’s instructions. The DNA content was determined by measuring the deoxyribonucleic acid (DNA) content using fluorophotometry with Hoechst 33258 (Sigma).

### 4.12. Quantification of Calcium Content

After bioreactor cultivation *in vitro*, cell-hydrogel constructs were harvested, lyophilized and weighed. Specimens were homogenized in 6 N hydrochloric acid, vigorously vortexed, and the supernatant collected for calcium content determination by colorimetric assay with cresolphthalein complexone (Sigma). Total calcium was expressed as a percentage of dry weight.

### 4.13. Real-Time PCR Analysis

Cell-laden scaffolds with chondrogenic medium were analyzed by real-time PCR using SyBr green system to investigate temporal mRNA expression changes of type I collagen (COL I) and osteopontin. The extraction of total mRNA was done following the reported protocols. The reaction mixture was kept at 95 °C for 10 min, followed by real-time PCR of 40 cycles. Each cycle included denaturation at 95 °C for 20 s, followed by annealing and extension at 61 °C for 1 min. The primers are shown in Table 1. Glyceraldehyde phosphate dehydrogenase (GAPDH) was the internal control. The comparative CT method was used for gene expression quantification.

**Table 1:** Sequences of primers used in real-time polymerase chain reaction.

Gene Symbol	Primer Sequence (5ʹ→3ʹ)		*T*_m_ (°C)
GAPDH	F:	5′-GAGCTGAACGGGAAACTCAC-3ʹ	67.8
	R:	5′-GGTCTGGGATGGAAACTGTG-3ʹ	
Collagen I	F:	5′-GATGGTCAGCCTGGACACA-3ʹ	67.8
	R:	5′-CGAAGGCCAGCAGGTCCAA-3ʹ	
Osteopontin	F:	5′-CAGTGGCTCAGCACCTGAA-3ʹ	67.8
	R:	5′-CGGCTCGATGGCTAGCTT-3ʹ	

F, Forward; R, Reverse; *T*_m_, Melting Temperature.

### 4.14. Histology Staining

The specimens were harvested and fixed in 4% paraformaldehyde solution (Sigma) at 4 °C overnight and then stored in 70% alcohol at 4 °C. The specimens were embedded in paraffin, sectioned to 5 μm, and processed for hematoxylin and eosin staining to distinguish osseous tissue phenotypes. Calcium deposition of the tissue constructs were visualized by Alizarin Red S staining. The sections were histologically viewed on a light microscope (Olympus, Tokyo, Japan).

### 4.15. Immunofluorescence Staining

For immunofluorescence staining, LF cells cultivated on chamber slides were fixed in 10% neutral buffered formalin. Following permeabilization with 0.2% Triton X 100 (USB Corp., Cleveland, OH, USA) and blocking solution treatment (5% non-fat milk in PBS with 0.1% Triton X 100) for 30 min at room temperature, the slides were incubated with mouse anti-porcine mono-clonal primary antibodies of type I collagen, osteocalcin, and osteopontin were applied for 1.5 h at room temperature. Subsequent to being washed three times with 1% non-fat milk in PBS with 0.1% Triton X 100, the slides were treated with Alexa 488 anti-mouse immunoglobulin G1 secondary antibody (Invitrogen Life Technologies, Carlsbad, CA, USA) for 45 min at room temperature. Finally, the samples were mounted using mounting solution with DAPI (Invitrogen) and left to dry overnight prior to observation.

### 4.16. Statistical Analysis

The data of gene expression results were presented as means ± standard deviation (SD), with three experiments (*n* = 3) for each test. The control and experimental groups were compared using the *t*-test.

## 5. Conclusions

In this study, we found that LF cells have osteogenic activity *in vitro* and *in vivo*. BMP-2 could improve the LF cells undergoing better bone formation *in vivo*. The PEGDA-BMP-2 hydrogel has the advantage of good bioavailability, non-toxicity, and absorbability. Different compositions of gel may affect not only the LF cells but also BMP-2 *in vivo* biologic activity. The true influence of gel could not be quantified or qualified easily. It is important to compare the biologic effect of different gels in further studies.
